# Amphotericin B Loaded Polymeric Nanoparticles for Treatment of *Leishmania* Infections

**DOI:** 10.3390/nano10061152

**Published:** 2020-06-12

**Authors:** Mudassara Saqib, A. Shabbir Ali Bhatti, Nasir M. Ahmad, Naveed Ahmed, Gul Shahnaz, Noureddine Lebaz, Abdelhamid Elaissari

**Affiliations:** 1 Department of Pharmacology and Therapeutics, Shaikh Zayed Postgraduate Medical Institute and Shaikh Zayed Medical Complex, Lahore 54000, Pakistan; 2 Department of Pharmacology and Therapeutics, Shalamar Medical and Dental College, Lahore 54000, Pakistan; shabbiralibhatti@gmail.com; 3 Polymer Research Lab, School of Chemical and Materials Engineering (SCME), National University of Sciences and Technology (NUST), H-12 Sector, Islamabad 44000, Pakistan; nasir.ahmad@scme.nust.edu.pk; 4 Department of Pharmacy, Quaid i Azam University, Islamabad 45320, Pakistan; natanoli@qau.edu.pk (N.A.); gshahnaz@qau.edu.pk (G.S.); 5 Univ Lyon, University Claude Bernard Lyon-1, CNRS, LAGEPP UMR-5007, 43 boulevard du 11 novembre 1918, F-69100 Villeurbanne, France; noureddine.lebaz@univ-lyon1.fr

**Keywords:** Amphotericin B, anti-leishmanial, anti-fungal, nanoprecipitation, drug delivery, polycaprolactone

## Abstract

Fungal infections in immune-compromised patients are an important cause of mortality and morbidity. Amphotericin B (Amp B) is considered a powerful fungicidal drug but its clinical usage has certain limitations when administered intravenously due to its toxicity and poor solubility. In consideration of such challenges, in cutaneous leishmaniasis, the topical application of Amp B can be a safer option in many aspects. Thus, herein, biopolymer of polycaprolactone (PCL) nanoparticles (NPs) were developed with the loading of Amp B by nanoprecipitation for the treatment of topical leishmanial infections. Various parameters, such as concentration of PCL and surfactant Poloxamer 407, were varied in order to optimize the formation of nanoparticles for the loading of Amp B. The optimized formulation exhibited a mean hydrodynamic particle size of 183 nm with a spherical morphology and an encapsulation efficiency of 85%. The applications of various kinetic models reveal that drug release from nanoformulation follows Korsmeyer–Peppas kinetics and has a high diffusion exponent at a physiological pH of 7.4 as well a skin relevant pH = 5.5. The activity of the prepared nanoparticles was also demonstrated in *Leishmania* infected macrophages. The measured IC_50_ of the prepared nanoparticle formulation was observed to be significantly lower when compared to control free Amp B and AmBisome^®^ for both *L. tropica KWH23* and *L. donovani* amastigotes in order to demonstrate maximum parasite inhibition. The prepared topical nanoformulations are capable of providing novel options for the treatment of leishmaniasis, which can be possible after *in vivo* assays as well as the establishment of safety profiles.

## 1. Introduction

Leishmaniasis is a protozoal disease initiated by a parasite of genus *Leishmania* and mostly caused by sand flies acting as vectors for transmission. It is a major health concern throughout the world. Currently, infected people total 12 million while annually around 1–2 million new cases are reported and could be fatal or self-healing [1]. The different infectious types of leishmaniasis are as follows: (a) cutaneous leishmaniasis; (b) mucocutaneous leishmaniasis; and (c) visceral leishmaniasis. Exposed body parts are mainly targeted by cutaneous leishmaniasis, while systemic leishmaniasis badly affects the internal organs of the body, including the spleen and liver. Multiple ulcers resulting from multiple bites by the sand fly are prevalent in cases throughout the world. Although cutaneous and visceral leishmaniasis represent major threats around the globe, mucocutaneous leishmaniasis is rarely reported [2]. A cure for cutaneous leishmaniasis (CL) exists in the use of prevalent antimonial modalities demanding different infusions with irregular sustainability and different reactions.

Amphotericin B (Amp B) is a second line leishmaniasis treatment which induces the eventual death of the parasites by its release in intracellular parts. Amp B exhibits physicochemical properties, such as a low molecular weight, low melting point and sufficient lipophilicity [3], which make it appropriate for topical delivery. Amp B is commonly administered intravenously as a leishmanial and anti-fungal agent which is associated with nephrotoxicity [4]. In cutaneous leishmaniasis, the topical application of Amp B might be a safer tactic. Moreover, there are also numerous other treatments, such as prime therapy, including meglumine antimoniate (Glucantime), but these are detrimental to some extent, requiring prolonged parenteral administration courses [5].

Nanotechnology has been widely utilized for drug delivery and to encapsulate various ingredients by multiple approaches including supercritical fluid technology, solvent diffusion methods, solvent evaporation, microemulsion, nanoemulsion, controlled and interfacial polymerization and nanoprecipitation [6]. For the encapsulation of hydrophobic drugs, emulsification techniques are recurrently stated and the nanoparticles are being developed using evaporation techniques [7]. For the improved outcomes of therapeutic regimens, polymeric nanoparticles have gained major attention due to their affinity with skin structure. It has been reported that the methods of the preparation and composition of polymers have a significant impact on encapsulation efficiency and particle size [8]. Nanocarrier-based topical drug delivery systems can be capable of overcoming various challenges associated with oral and parenteral administration routes, such as inefficient or low solubility drugs, and optimizing delivery within a desirable duration.

There is a need to develop novel drugs to counter leishmaniasis due to the existence of various hazards, including the high cost of current medicines [9], along with their possible toxic effects [3], and resistance development in parasites [10]. An appropriate topical formulation must be capable of targeting the *Leishmania* parasites in the dermal layers of the skin. Therefore, carriers are decisive in improving drug penetration into the skin and supporting drug release. In comparison to old-fashioned formulations, chemical permeation enhancers (CPEs) based on polymeric nanocarriers interact with the outermost components of the skin and the rate-controlling layer stratum corneum (SC), increasing its permeability and being retained longer at the site of administration [11]. Dimethylsulfoxide (DMSO) is one of the initial and most extensively studied chemical permeation enhancers (CPEs) and is frequently used in numerous areas of pharmaceutical science as a “universal solvent”. The interface of DMSO with lipids is believed to be significant in its enhancing action. It has been anticipated that DMSO could encourage lipid fluidity by disrupting the organizational structure of the lipid chains, which improves the diffusion transport of solutes [12].

**Figure 1:**
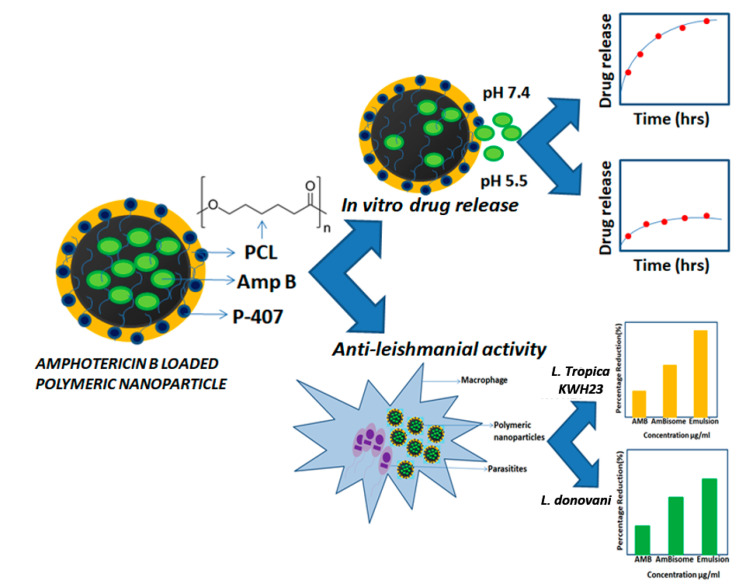
Overview of the experimental work: From Amphotericin B (Amp B) loaded polymeric nanoparticles preparation to in vitro drug release and anti-leishmanial activity.

The main objective of this study is the development of polymeric nanoparticles by nanoprecipitation through high-pressure homogenization for the treatment of leishmaniasis. These formulations are desirable to reduce the side effects specifically associated with the oral route of administration. The purpose of using the topical route was to resolve the challenges associated with the low solubility and poor absorption of the drug when administered through the oral route. To accomplish the desired objectives, a combination of high-pressure homogenization (HPH) and solvent diffusion techniques was used to fabricate nanoparticles. Particles of smaller sizes were obtained through the HPH technique, which may be very helpful for topical drug delivery. Furthermore, to the best of our knowledge, Amp B nanoparticles have not previously been formulated and explored in detail using polycaprolactone (PCL) as the only ingredient. Polycaprolactone is a biodegradable polymer used for the delivery of various active moieties through different routes and especially for topical drug delivery [13,14,15]. This approach is able to eliminate the use of relatively scarce and costly ingredients, overcoming economic issues. This method can also provide better drug loading and improved entrapment efficiency. An overview of the experimental work and observations are presented schematically in Figure 1 in order to elaborate the preparation of the polymer nanoparticles with efficient Amp B drug loading and in vitro studies of pH dependent release and anti-leishmanial activities against *L. topicana KWH23* and *L. donavini*.

## 2. Materials and Methods

### 2.1. Materials

The United States Pharmacopeia (USP) grade Amp B was acquired from Synbiotics, Vadodara, Gujarat, India. Dimethylsulphoxide (DMSO), polycaprolactone (PCL), Poloxamer 407, monobasic potassium phosphate, sodium hydroxide (NaOH) and Sabouraud dextrose agar (SDA) were obtained from Sigma Aldrich, Humberg Germany. *Leishmania tropica* KWH23 (*L. tropica*) and unicellular parasite *Leishmania donovani* (*L. donovani*) strains were obtained from the fungal culture bank of Pakistan (FCBP) and maintained on SDA at 4 °C prior to use. During the experimental work, deionized water with a resistivity of 18.2 MΩ.cm (at 25 °C) was used to prepare all the solutions. Tissue culture slides (NUNC^®^; Thermo Fisher Scientific^®^, Waltham, MA, USA) were used to study the anti-leishmanial activities of the prepared nanoparticle formulations.

### 2.2. Methods

#### 2.2.1. Preparation of Solutions

For the preparation of the organic phase for blank emulsion, 10 mg of PCL was dissolved in 1 mL of DMSO and the final volume was made up to 5 mL followed by sonication in a bath sonicator until completely dissolved. For drug-loaded polymeric nanoparticles, the organic phase was prepared by dissolving 10 mg of PCL in 1 mL of DMSO and the final volume was made up to 5 mL and sonicated in the bath sonicator until completely dissolved. Then, 5 mg of Amp B was added with continuous stirring until complete dissolution and the final volume was made up to 5 mL.

The nanoprecipitation method was used for the preparation of the nanoparticles with slight modification. A high-pressure homogenization technique was used for this purpose and 10 mL of 2% Poloxamer solution was placed at 6000 rpm with continuous stirring. The organic phase (5 mL) was taken into a syringe and injected slowly into a surfactant solution at a constant rate of 0.25 mL/min. The formulation was left in open air overnight so the organic phase could be eluted in the aqueous phase. Centrifugation was performed at 10,000 *g* for 30 min at 30 °C to in order to attain nanoparticle pellets.

For the optimization of the prepared formulation of PCL nanoparticles, various parameters, such as polymer concentration, organic and aqueous phase ratio and surfactant concentration, were studied.

#### 2.2.2. Formulation of Drug-Loaded Emulsion

The same procedure was followed to prepare the drug (Amp B) loaded polymeric nanoparticles, and the only difference was in the organic phase. In this formulation, the organic phase contained Amp B that had already been dissolved, which was injected into the surfactant solution at a constant rate. Optimization of the formulation was carried out by changing the polymer, organic and aqueous phase ratio and surfactant concentration.

### 2.3. Characterization Techniques

The prepared nanoparticles and formulations were thoroughly characterized using different techniques to explore their size, size distribution, morphology and surface charge. A high-resolution scanning electron microscope (TESCAN VEGA-3, MODEL IMU VP-SEM, New York, NY, USA) was used to analyze the size and surface morphology. Particle size analysis (Nano Zetasizer (ZS), Malvern Instruments, Malvern UK) was carried out to determine the effect of various parameters on the formulation of the emulsion. The particle size distribution and surface charge of the blank and drug-loaded nanoemulsions were analyzed in the diluted form with the help of dynamic light scattering (Zetasizer). The results were obtained by repeating the method thrice and the mean value was acquired in order to obtain both the particle size distribution and the polydispersity index (PDI) of the prepared nanoparticle formulations. A UV-visible spectrophotometer (Dynamica, Halo DB-20, Livingston, UK) was used to evaluate the amount of drug encapsulated in the polymeric nanoparticles. The standard curve of the drug was established and, based on this, drug loading and release studies were carried out.

### 2.4. In Vitro Drug Release Studies and Release Kinetics

A drug release study of the prepared formulations was carried out at pH = 7.4 and pH = 5.5. A volume of 10 mL of the formulation in a dialysis bag was added to 50 mL of PBS solution that was maintained at 37 °C on a shaking water bath for estimation of the drug release. A volume of 2 mL of the PBS solution was taken out after definite time intervals from 0.25 to 48 h and analyzed through a UV-visible spectrophotometer at a 408 nm wavelength. The same amount of PBS solution was added to compensate for the solution that was withdrawn. The drug release profiles were compared at both pH values. The encapsulation efficiency was calculated by centrifugation of the formulation for 1 h at 10,000 *g* at 30 °C. Before centrifugation, the formulation (1 mL) was taken into a falcon tube and the volume was made up with DMSO up to 10 mL. For the determination of the drug release and mass transport mechanism, various kinetic models, such as zero-order, first-order, Higuchi and Korsmeyer–Papas, were applied [16]. Application of these models predicted the drug release mechanism for the Amp B loaded nanoparticles.

### 2.5. In Vitro Anti-Leishmanial Activities

An amastigote model in a macrophage cell line was used to evaluate the anti-leishmanial activity of the developed formulations. For this purpose, the J774 cells were resuspended (2.5 × 10^5^ cells/mL) in an RPMI-1640 culture medium without serum. The cells were plated onto 8-well Lab-Tek CCR2 tissue culture slides at a density of 200 × 10^3^ cells/well and incubated at 37 °C for 24 h in a humidified incubator. The cells were then washed twice with a serum-free medium and infected with 100 μL metacyclic stage of *L. tropica KWH23* at an infection ratio of 10:1 (parasites/macrophages) in 200 μL of the whole medium (RPMI 1640 + 10% heat-inactivated fetal calf serum + 50 mg/L gentamicin), and then they were incubated for 12 h. Non-phagocytosed parasites were removed by washing three times with PBS and the wells were supplemented with a RPMI-1640 complete medium. Stock solutions of native Amp B and emulsion were prepared in 100% DMSO at 1 mg/mL Amp B formulations available commercially as AmBisome^®^. The Amp B formulations were reconstituted consistent with the manufacturer’s protocol in order to achieve a 5 mg/mL stock of Amp B emulsion. Working concentrations were prepared in the whole medium (RPMI 1640 + 10% heat-inactivated fetal calf serum + 50 mg/L gentamicin). The cells were treated with emulsion and Amp B formulations at six different drug concentrations (1–0.004 μg/mL Amp B), prepared by serial dilution. Untreated infected macrophages were used as positive controls. Each formulation concentration was tested in quadruplicate.

Statistical analysis was also performed using the unpaired, two tailed t-test with the significance threshold set at * *p* < 0.05 and ** *p* < 0.01, which served as the cutoff level (α). If the p-value was less than α, then this was considered to be significant for all analyses. Three statistical differences were calculated to determine the *p* values: p1 was between Amp B/AmBisome^®^, p2 was between AmBisome^®^/emulsion and p3 was between Amp B/emulsion. If the difference was lower than the threshold (* = *p* < 0.05 and ** = *p* < 0.01), this meant that the difference was significant and hence the results were indeed true in terms of creating an effect. Error bars show the standard deviation and asterisks (* or **) represent significant p-values.

## 3. Results and Discussion

The development of polymeric nanoparticles by nanoprecipitation was carried out through high-pressure homogenization for the treatment of leishmaniasis. The formulation was supposed to reduce the side effects specifically associated with the oral route of administration. The purpose of using the topical route was to treat local infections such as cutaneous leishmaniasis and to avoid high systemic concentrations that cause side effects such as nephrotoxicity.

**Figure 2:**
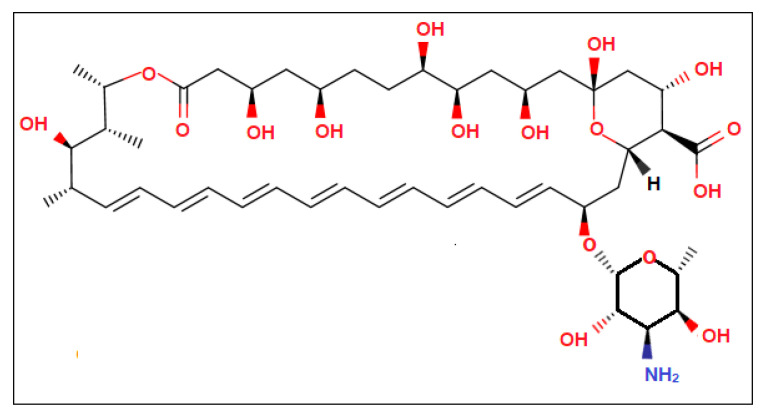
Chemical structure of Amp B representing its functional groups.

Amp B is one of the key drugs utilized for the treatment of fungal infections and leishmaniasis, though poor bioavailability and gastrointestinal irritation may lead to reduced effects and patient non-compliance. The structure of Amp B, as shown in Figure 2, indicates that it acts by binding to sterols present in the cell membrane of vulnerable parasites that change the permeability of the membrane. Among various biopolymers, polycaprolactone (PCL) has exhibited superior properties in terms of sustained release, enhanced loading capacity and higher *in vivo* absorption of encapsulated drugs [17]. Poloxamer 407 (P-407), a non-ionic surfactant, possesses the highest solubilization capacity and the lowest toxicity compared to polyoxyethylene sorbitan monolaurate (Tween 20) [18]. It has also been proposed that DMSO may intermingle with membrane proteins, leading to organizational defects at the intercellular keratin protein in the stratum corneum–lipid border, which may heighten its permeability. Hence, it is interesting to study the potential of DMSO and Poloxamer 407 to enhance skin permeation of Amp B when supplemented into a polycaprolactone polymeric nanoemulsion.

### 3.1. Optimization and Stability of the Formulations

For the optimization of the formulations, the polymeric nanoparticles were synthesized by varying concentrations of surfactant (Poloxamer 407), polymer (PCL) and organic (DMSO) or aqueous solvents. All the prepared emulsions were evaluated on the basis of their particle size and physical stability. The physical appearance and stability status at the ambient temperature of the prepared polymeric nanoparticle formulations are presented in Table 1, along with their mean sizes and polydispersity index (PDI) values. It can be observed that for the stable formulation, an optimized range of polymer or surfactant concentrations is required.

A preliminary study which varied the amount of PCL in the aqueous phase at a fixed amount of oil and surfactant showed that a minimum of approximately 0.05% was required for a suitable consistency. The different concentration of the polymer was 10 to 50 mg in the solution. Particle size and physical stability were prominently affected by low and high concentrations of the polymer. Low concentration of PCL produced particles of smaller sizes while for high concentrations, particle size was increased. Higher PCL concentrations (FK-5) were observed to produce unstable nanoparticle formulations and found to be relatively thicker with aggregates. The stability and consistency of these formulations were generally lost within two days and also resulted in a globular appearance. The formulation with a PCL concentration ranging from 10 to 40 mg resulted in fairly good stability in terms of appearance and absence of phase separation for at least 30 days. The prepared nanoparticle formulation’s characteristics, such as particle size and physical stability, were noticeably affected by the concentration of PCL. A low concentration of PCL produced particles of smaller sizes while for a high concentration, particle size was increased, as observed, respectively, in the FK-1 to FK-5 cases. The results showed that the size of the nanoparticles depended on the polymer concentration, because polymers have the tendency to coalesce at high concentrations [19]; alternatively, this could be due to density differences between the external and internal phases, or it may have occurred due to the reduced diffusion rate of the solute molecules in the outer phase [20]. The different concentrations of surfactant (from 0.5 to 2.5%) were analyzed. It was revealed that the optimum surfactant concentration for a stable formulation was between 0.5% and 2%, as shown in Table 1. The prepared formulations resulted in uniform consistency which remained stable for more than 30 days. An increasing amount of surfactant resulted in relatively unstable particles, as observed in the case of FK-10. A low concentration of surfactant produced particles of larger sizes, while high concentrations reduced the particle size. An increment in particle size with an increase in surfactant concentration might be due to a reduction in surface tension between the organic and aqueous phases. The surfactant also prevents the aggregation of particles and stabilizes the nanoparticles [12]. FK-9 with 2% surfactant, an organic to aqueous ratio of 1:2 (5 mL DMSO) and 10 mg of PCL was used as an optimized formulation for drug loading and further analysis. This was selected on the basis of the data presented in Table 1, as the formulation was physically stable with a smaller particle size (167 nm).

### 3.2. Size and Morphology of Prepared Polymeric Nanoparticles

**Figure 3:**
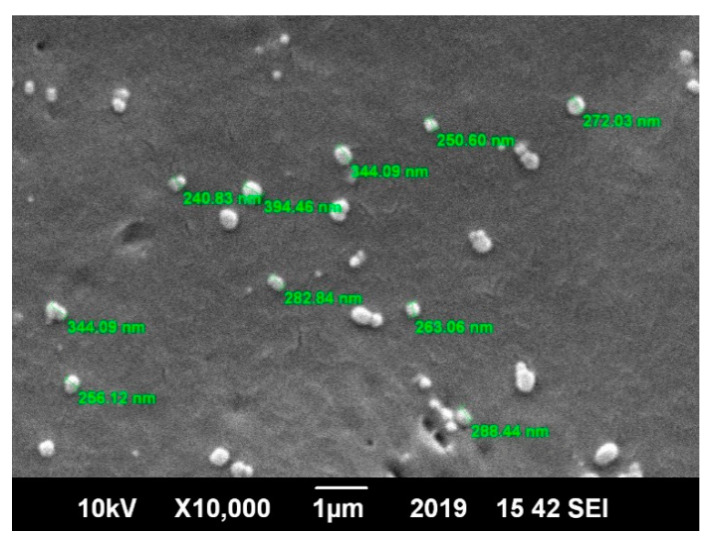
Scanning electron microscope (SEM) image of the polycaprolactone (PCL) polymer nanoparticles with spherical morphology and loaded with the Amphotericin B drug.

Scanning electron microscopy (SEM) was utilized to determine the size and morphology of the blank and drug-loaded nanoparticles. The SEM image in Figure 3 shows that the prepared nanoparticles were spherical in shape and spatially separated, which confirmed the absence of aggregation. The nanoparticles were uniform in size and shape. This gave a preliminary result about the broadness of the particle size distribution (low polydispersity), which was in excellent agreement with previous studies [21].

### 3.3. Surface Charge and Particle Size Distribution

**Figure 4:**
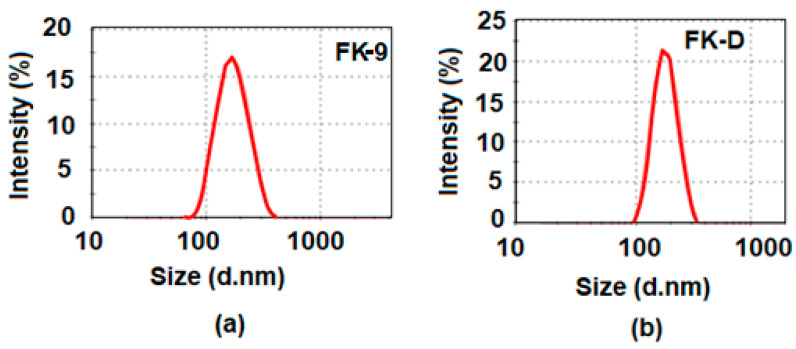
Particle size distributions of (**a**) blank optimized nanoemulsion (FK-9) and (**b**) Amp B drug-loaded formulation (FK-D) in PCL polymer nanoparticles.

The particle size distribution of the prepared blank and Amp B encapsulated formulations are given in Figure 4a,b, respectively. The average size of the blank and drug-loaded nanoparticles was 167 nm and 183 nm, respectively. The polydispersity index (PDI) determined the homogeneity of the nanoparticles, which was found to be 0.211 (in the case of Amp B loaded nanoparticles) and thus indicated uniformity in the size and homogeneity in the size distribution of the prepared nanoparticles. In general, when the PDI value is less than 0.1, it indicates the occurrence of a monodispersing system, while PDI values in a range of 0.1–0.4 and more than 0.4 describe moderate and high polydispersity aspects of the distribution, respectively [22]. The size of the nanoparticles ranged from around 80 to 300 nm in the case of the Amp B loaded nanoparticles, as shown in Figure 4b, and between 100 to 200 nm for the blank nanoparticles, as presented in Figure 4a. This reflects the narrow aspect of the size distributions. This observation is in good agreement with the SEM analysis and indicates the stability of the suspensions and the absence of aggregation.

The excellent stability of the polymeric nanoparticles prepared by the high-pressure homogenization method can be attributed to their quasi-neutral charge, since the zeta potential was almost zero for all the samples, as reported in Table 1. This is due to the ability of Poloxamer to reduce the low repulsive electrostatic charge of PCL nanoparticles. The stability within all the formulations is then due to the presence of Poloxamer around the PCL nanoparticles that provides steric stabilization for the obtained colloidal dispersions. For the stable dispersion, the colloidal stability can be attributed to sterical stability in the case of low Poloxamer amount, and in the case of moderate Poloxamer amount, the colloidal stability can be attributed to depletion stabilization. Contrastingly, for high amounts of Poloxamer, the observed instability is due to the depletion aggregation of the formed particles.

Moreover, the zeta potential of all samples was found to be close to zero and the zeta potential of sample FK-9 has been measured as a function of pH ranging from 3 to 10. The observed values were found to be around zero, irrespective of pH variation. This highlights the screening effect of the low PCL-based particles by non-charged Poloxamer and thus corroborates the above mentioned observation.

### 3.4. In Vitro Drug Release Study

**Figure 5:**
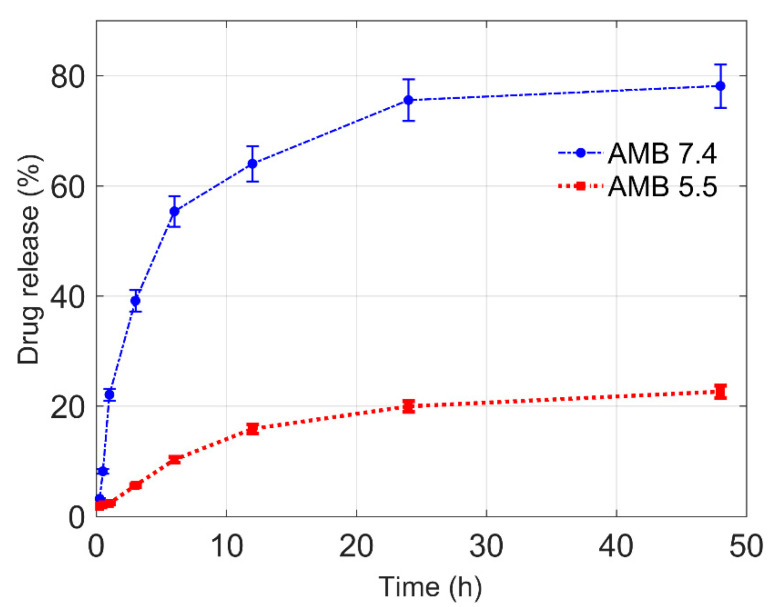
Amp B drug release profile from the prepared polymeric nanoparticle in phosphate buffer at different pH values of 7.4 and 5.5.

The drug release studies of Amp B encapsulated in polymeric nanoformulations was performed by the diffusion method, using a dialysis bag for a period of 48 h in pH = 7.4 and pH = 5.5 phosphate buffer solutions maintained at 37 °C using a water bath. A graphical representation of cumulative drug release (in percent) versus time plots at pH = 7.4 and pH = 5.5 is shown in Figure 5. From the graphs, it can be observed that there was a persistent drug release from the nanoformulation at pH = 7.4 and approximately 78% of the encapsulated drug was released within 48 h. However, in the case of pH = 5.5, only 22% of the drug was released, which shows reduced permeation through the nanoparticles. The in vitro release of Amp B from the polymer at pH = 7.4 was found in a sustained manner due to the cleavage of ester linkages of PCL [23].

The in vitro release study indicated the pH-dependent release profile of the drug, showing the insignificant amount of drug released in the acidic medium (pH = 5.5) as compared to the drug released at around a neutral medium (pH = 7.4). A continuous drug release was observed at pH = 7.4 as compared to pH = 5.5, showing that pH has a strong influence on the release kinetics of Amp B from the polymer matrix. A higher drug release at pH = 7.4 reveals a favorable interaction between Amp B and the release neutral medium. Polymer-drug interaction, drug solubility in the medium and polymer interaction with the release medium must also be considered in order to understand the drug release kinetics [24]. The lower release at pH = 5.5 could be interpreted as a more favorable interaction between drug and polymer than drug and release medium. This can be explained on the basis of the dissolution behavior of the polymer nanoparticles loaded with Amp B in varied pH conditions. It is possible that for the prepared nanoparticles, a relatively dense polymer chain structure originates when particles interact with an acidic medium, but in relatively neutral conditions or a higher pH, Amp B easily leaches out from the particle due to the relatively less dense or more porous structure. It should also be considered that in general polyesters such as poly (glycolide), poly (lactide) and polycaprolactone (PCL) or their copolymers have been used for drug delivery applications [25]. In the case of PCL, the release of drugs can be incomplete because of its higher crystallinity and hydrophobicity [26]. In consideration of such challenges, the design and development of drug delivery systems based on the blending of PCL with other polymers or its copolymers can be considered in principle to improve the control release of drugs at various pH levels and to tune the permeability of PCL for achieving a desirable delivery [27].

The mechanism of Amp B release and the kinetics order of drug release from the polymeric nanoparticles were studied by fitting the in vitro drug release data of the formulation at different pH into different kinetic models, which were the zero-order, first-order, Higuchi and Korsmeyer–Peppas models [13]. Zero-order release kinetics describe systems where the drug release rate is constant over a period of time and independent of the concentration of drug in the polymeric system (Equation (1)) [28]:

(1) $$M_{t}=M_{\infty }+kt$$

where *M_t_* is the absolute cumulative amount of drug released at time *t*, *M*_∞_ is the absolute cumulative amount of drug released at infinite time and *k* is the constant of the considered system.

A first-order model describes a system in which the drug release from the polymer matrix is influenced by the external drug concentration. Its general equation is given as [28]:

(2) $$\ln\left(\frac{M_{t}}{M_{\infty }}\right)=kt$$

The Higuchi model describes the release of a drug from porous matrices as the square root of the time dependent process, based on Fickian diffusion [29]. It was derived under pseudo-steady state assumptions and it is given in its simplest form as:

(3) $$\frac{M_{t}}{M_{\infty }}=K\sqrt{t}$$

The Korsmeyer–Peppas model is a generalization of the Higuchi model and describes drug release from the polymeric system as a not fully known release mechanism, and hence release data are fitted and described as [30]:

(4) $$\ln\left(\frac{M_{t}}{M_{\infty }}\right)=\ln\left(K\right)+n\ln\left(t\right)$$

where *n* is the drug release exponent or diffusion exponent. It is worth noting that for *n =* 1/2, the Korsmeyer–Peppas model is equivalent to the Higuchi model.

Experimental kinetic release data were fitted using the four different models by a least-square minimization algorithm and the R-squared (R^2^) values of the different cases are summarized in Table 2.

The R-squared value ranges from zero to 1 and provides information on the quality of the regression. For a model that perfectly fits the experimental data, this indicator is equal to unity. Note that the first three models give non-satisfactory fits since R-squared is far from unity, whereas in the case of the Korsmeyer–Peppas model, the good choice of diffusion exponent (n) leads to the very good fit of the experimental data for the two different pH conditions. The diffusion exponent (n) values of Korsmeyer–Peppas plots are 0.499 and 0.694 at pH *=* 7.4 and pH *=* 5.5, respectively.

**Figure 6:**
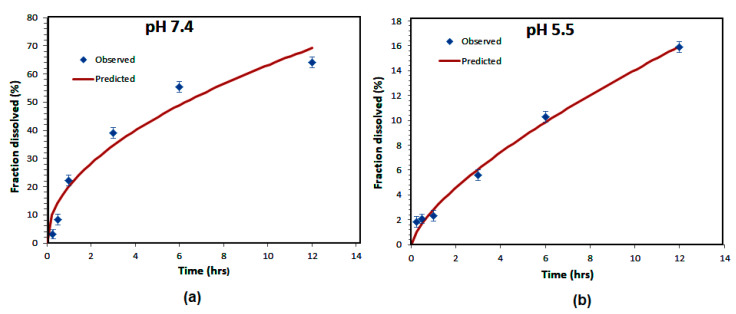
Korsmayer–Peppas kinetic models of Amp B release from polymeric nanoparticles at pH *=* 7.4 (**a**) and pH *=* 5.5 values (**b**).

The value of n is very useful and provides information about the physical mechanism controlling the drug release from the particles. Based on the value of this exponent, the drug release was controlled by non-Fickian (anomalous) transport at both pH levels [31]. Data analysis using all mathematical models reveals that drug release from the nanoparticles follows a Korsmeyer–Peppas release kinetics with maximum R^2^ values and high diffusion exponents at both pH levels, as presented in Figure 6.

### 3.5. Encapsulation Efficiency

The encapsulation efficiency (EE%) is the percentage of drug that is successfully entrapped into the polymeric nanoparticles. The encapsulation efficiency of the Amp B loaded nanoparticles was analyzed through a UV-visible spectrophotometer and found to be approximately 86% using Equation (5). The higher EE% enables researchers to deliver the drug at a higher dose, more precisely at the site of the action. The use of PCL enables them to enhance the nanoparticles in order to entrap drug molecules, and it also enhances aqueous solubility, promoting drug escape from the nanoparticles [23]. As compared to the solvent emulsification method, the use of the HPH technique in the present work led to a higher encapsulation efficiency [21].

(5) $$Encapsulation\;Efficiency\;\left(EE\%\right)=\frac{Total\;drug\;added-Drug\;found\;in\;supernatant}{Total\;drug\;added}\times 100$$

### 3.6. Pharmacological Evaluation of Anti-Leishmanial Activities

**Figure 7:**
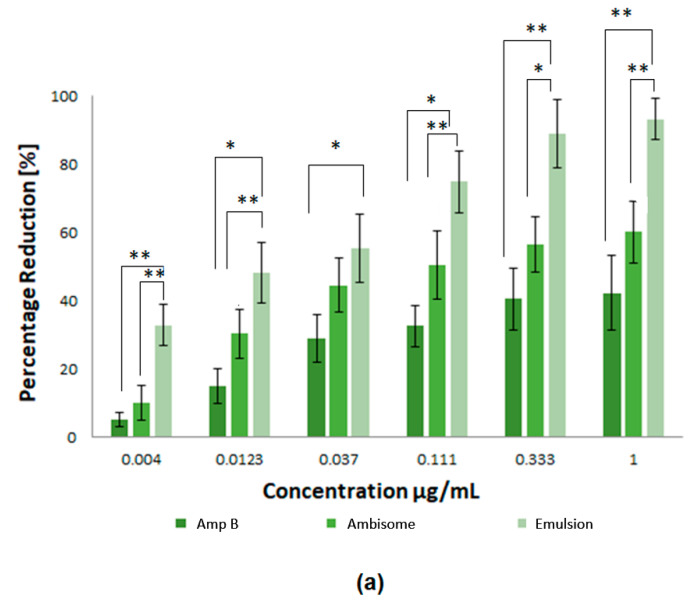

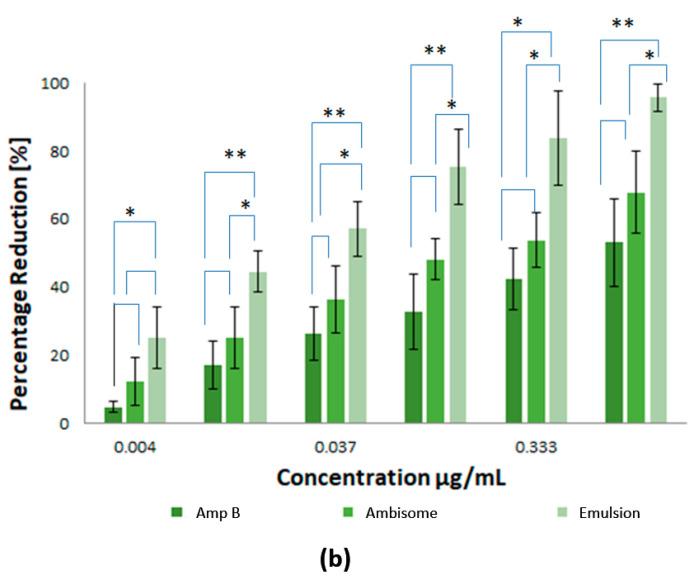
Pharmacological evaluation of the anti-leishmanial activities of the polymeric nanoparticles where different concentrations of nanoformulations were utilized: (**a**) inhibition of *L. tropica* KWH23 amastigotes at various concentrations and (**b**) inhibition of *L. donovani* amastigotes at various concentrations. Results are presented as mean ± SD of four experiments and were analyzed by paired t test and with a significance threshold denoted by *p* values set at * = *p* < 0.05 and ** = *p* <0.01.

For the efficiency of potential drugs against *Leishmania*, as an intracellular parasite, it is essential that the drug is able to access the amastigote forms of the parasites inside their host cells. In consideration of this, the activity of the prepared nanoparticles was determined in *Leishmania* infected macrophages. In the current study, the Amp B formulations were prepared with different concentrations and investigated against *L. tropica* KWH23 and *L. donovani* amastigotes in a concentration dependent manner. Free Amp B and AmBisome^®^ (a commercially available marketed formulation) were used as controls. Figure 7 represents a pharmacological evaluation of the anti-leishmanial activities of the polymeric nanoparticles, which are also compared with Amp B and AmBisome^®^ at different concentrations (1–0.004 μg/mL Amp B) as prepared by serial dilution. It was obvious that the prepared emulsion loaded with Amp B significantly improved its anti-lesihmanial activitiy. As seen in Figure 7a,b, the greatest mean percentage inhibition of the *L. donovani* amastigotes, mediated using the prepared nanoformulations at different concentrations, was achieved by using the emulsion, followed by AmBisome® and then Amp B, which showed the least amount of inhibition. For statistical analysis, the difference in mean percentage at different formulation concentrations for 0.004, 0.0123, 0.037, 0.111, 0.333 and 1µg/mL were tested for significance using the unpaired, two tailed t-test, with the significance threshold set at * *p* < 0.05 and ** *p* < 0.01. The difference in p1 between Amp B and AmBisome^®^ was significant for few formulation concentrations values while the difference in p3 between AmBisome^®^ and the emulsion was significantly different for many formulations’ concentration values. However, the difference between Amp B and the emulsion was found to be significant for all values of the formulation concentrations.

Exposure of the parasites to Amp B, AmBisome^®^ and the emulsion demonstrated that all the prepared samples were able to inhibit parasite growth. The measured half maximum inhibitory concentration (IC_50_) of Amp B, AmBisome^®^ and emulsion for *L. tropica* KWH23 was found to be 0.256 ± 0.09, 0.19 ± 0.05 and 0.03 ± 0.009 μg/mL, respectively; and for the *L. donovani* amastigotes these values were 0.289 ± 0.07, 0.21 ± 0.05 and 0.023 ± 0.007 μg/mL, respectively. Macrophage targeting through drug loaded formulations significantly enhanced and improved the anti-leishmanial activity of Amp B for the inhibition of intracellular parasites. The prepared drug loaded formulation for anti-leishmanial activity against infected macrophages provided maximum parasite inhibition. The formulation with the low drug concentration was able to inhibit the intracellular replication of parasites as compared to clinically used AmBisome^®^. The IC_50_ of emulsion was lower than Amp B and AmBisome^®^, which was due to the enhanced anti-leishmanial activity. The emulsion reduced the number of parasites and the macrophages were free of parasites after treatment the with emulsion at a concentration of 1 μg/mL. Amp B and AmBisome^®^ were less effective than the prepared emulsion at reducing the intracellular parasites [32]. The interaction of PCL polymer with Amp B against *Leishmania* showed a synergistic effect with a twofold reduction of parasites, by enhancing the ability of Amp B to disrupt membrane function, as well as potential direct effects of Amp B on trypanothione reductase or mitochondrial function. The percentage inhibition of the *L. tropica* KWH23 and *L. donovani* amastigotes of the formulations were calculated by Equation (6), utilized at a concentration that was biocompatible with the macrophages [33].

(6) $$Inhibition\;\left(\%\right)=\frac{Number\;of\;cells\;in\;control\;well-Number\;of\;cells\;in\;treated\;well}{Number\;of\;cells\;in\;control\;well}\times 100$$

The free Amp B and Amp B loaded emulsion reduced the infection index in a dose dependent manner. The encapsulation of Amp B inside the polymer enhanced the oxidative damage activity of Amp B to destroy parasites. The maximum DMSO concentration of 0.1% was found to have no influence on macrophage/amastigote clearance. After 72 h of incubation (5% CO_2_ at 37 °C), slides were fixed with 100% methanol for 1 min and stained for 10 min with 10% Giemsa’s solution. The Giemsa-stained intramacrophage amastigote slides were visualized under a light microscope (Zeiss, Pleasanton, CA, USA). The percentage inhibition from the test formulations and the Amp B emulsion were calculated as cells/100 nucleated nontreated control cells. Data were fitted using the nonlinear dose-response sigmoidal curve, and the IC_50_ values were estimated by least-square regression fitting. Similarly, therapeutic efficacy evaluations of the developed nanoformulations against *L. donovani* were performed on the amastigote model in a macrophage cell line, as described above. It should be noted that blank formulations were not introduced in the assay as negative controls because it was expected that PCL and other related polymer-based formulations would have no significant effects or activity when used alone. This hypothesis was also supported by the already published literature discussing PCL-based as well as other polymer-based formulations of Amp B and for other drugs where no negative control of the polymeric nanoparticles was used, as discussed elsewhere [34,35,36]. Additionally, studies using PCL-based drug formulations, where polymeric nanoparticles were used as negative controls, reported no significant effects or activities of these negative controls, as reported for Sertaconazole [37]. The development of the formulation exhibited a substantial anti-microbial response and demonstrated its evident anti-leishmanial efficacy. The improved activity of the emulsion can be attributed to the targeted delivery of the therapeutic agent at the intracellular sites that serve as a reservoirs for parasites. The observed experimental results are significant and highlight the importance of further exploring the development and applications of nanoparticle-based therapeutics for the treatment of *Leishmania* infections.

## 4. Conclusions

Polyacaprolactone (PCL) nanoparticles loaded with Amp B were developed for topical application in Leishmaniasis infections. Parameter optimization through variation of the concentrations of PCL polymer and Ploxomor 407 surfactant at fixed DMSO solvent concentrations was carried out in order to prepare the nanoparticles, using high-pressure homogenization and solvent diffusion techniques. The average size of the optimized blank and drug-loaded nanoparticles was 167 and 183 nm, respectively. The lowest polydispersity index (PDI) was found to be 0.211 in the case of the Amp B loaded nanoparticles. The zeta potential of the prepared nanoparticles was found to be close to zero and did not appear to be affected by pH variations because of the possible screening effect of the PCL-based particles by the non-charged poloxamer. The in vitro release followed a Korsmeyer–Peppas release kinetics model, and a high diffusion exponent at a physiological pH of 7.4 as well at skin relevant pH = 5.5 was pointed out. The pH dependent release profile of the drug was observed to exhibit the lowest amount of drug released in the acidic medium (pH = 5.5), as compared to the higher drug released in the neutral medium (pH = 7.4). The encapsulation efficiency of the Amp B loaded nanoparticles was found to be 85.90%. The activity of the prepared nanoparticles was also demonstrated in *Leishmania* infected macrophages. The Amp B formulations were prepared with different concentrations (1–0.004 μg/mL) and investigated against *L. tropica KWH23* and *L. donovani* amastigotes in a concentration dependent manner. Free Amp B and commercially available AmBisome^®^ were used as controls. Exposure of the parasites to Amp B, AmBisome^®^ and the emulsion demonstrated that all the prepared samples were able to inhibit parasite growth. The measured IC_50_ of the prepared nanoformulations was observed to be significantly lower as compared to free Amp B and AmBisome^®^ for the *L. tropica KWH23* and *L. donovani* amastigotes. Macrophage targeting through drug loaded formulations significantly enhanced and improved the anti-leishmanial activity of Amp B for the inhibition of intracellular parasites. The prepared drug loaded formulation for anti-leishmanial activity against infected macrophages provided maximum parasite inhibition. The formulation with low drug concentrations was able to inhibit the intracellular replication of parasites as compared to clinically used AmBisome^®^. The prepared nanoformulations were able to provide novel options for the treatment of leishmaniasis, which will be possible after *in vivo* assays as well as the establishment of safety profiles.

## Figures and Tables

**Table 1:** Composition of different formulations used in the study.

Code	Polymeric Phase		Aqueous Phase	Mean Particle Size (nm)	PDI *	Zeta Potential (mV)	Observation
	Polymer (mg)	Solvent (ml)	Poloxamer 407 (%)				
FK-1	10	5	2.0	203	0.195	~0	Stable
FK-2	20	5	2.0	240	0.191	~0	Stable
FK-3	30	5	2.0	223	0.130	~0	Stable
FK-4	40	5	2.0	225	0.102	~0	Stable
FK-5	50	5	2.0	/	/	/	Unstable
FK-6	10	5	0.5	196	0.111	~0	Stable
FK-7	10	5	1.0	215	0.149	~0	Stable
FK-8	10	5	1.5	221	0.173	~0	Stable
FK-9	10	5	2.0	167	0.180	~0	Stable
FK-10	10	5	2.5	/	/	/	Unstable

* PDI: Polydispersity Index

**Table 2:** R^2^ values evaluated for kinetic modeling of in vitro drug release studies at pH values of 7.4 and 5.5.

pH of Release Medium	Zero-Order	First-Order	Higuchi	Korsmeyer–Peppas	Release Mechanism
7.4	0.027	0.812	0.776	0.944	Non-Fickian transport
				(*n* = 0.499)	
5.5	0.577	0.652	0.948	0.992	Non-Fickian transport
				(*n* = 0.694)	
